# Supplementary material for: Role of Inflammation in Depressive and Anxiety Disorders, Affect, and Cognition: Genetic and Non-Genetic Findings in the Lifelines Cohort Study
Source: medRxiv. 2024 Apr 19:2024.04.17.24305950. Preprint. [Version 1] doi: 10.1101/2024.04.17.24305950 (PMC11065023; doi:10.1101/2024.04.17.24305950)
Supplement: Supplement 1 [file media-1.pdf]

Supplementary Material

SUPPLEMENTARY LIFELINES GROUP AUTHOR INFORMATION 2

SUPPLEMENTARY METHODS 2

1.1. Cogstate Test Battery 2

1.2. Lifelines Genetics Data 3

1.3. Details of GWAS and Instruments used to create GRS in Lifelines 4

1.4. Adjustment for relatedness in genetic analyses 5

1.5. Creating Genetic Relationship Matrices (GRM) 5

SUPPLEMENTARY RESULTS 5

2.1. GRS analysis 5

2.2. GRSs associations with exposures and potential confounders 6

SUPPLEMENTARY DISCUSSION 6

3.1. Possible factors contributing to mixed MR results of CRP-depression 6

SUPPLEMENTARY TABLES 8

Table 1. Access to GWAS Data 8

Table 2. Details of GWAS used to create GRS 9

Table 3. Standardised GRS on potential confounders (GRAMMAR adjusted) 10

Table 4. Standardised GRS on standardised outcomes (GRAMMAR adjusted) 11

Table 5. Standardised GRS on binary outcomes (unadjusted analysis) 14

Table 6. Standardised GRS on continuous outcomes (unadjusted analysis) 15

Table 7. Standardised GRS on binary outcomes (1st degree relatives removed) 17

Table 8. Standardised GRS on binary outcomes (2nd degree relatives removed) 18

Table 9. Standardised GRS on binary outcomes (3rd degree relatives removed) 19

Table 10. Standardised GRS on continuous outcomes (1st degree relatives removed) 20

Table 11. Standardised GRS on continuous outcomes (2nd degree relatives removed) 22

Table 12. Standardised GRS on continuous outcomes (3rd degree relatives removed) 24

SUPPLEMENTARY FIGURES 26

Figure 1. Flow diagram of total sample size available for genetic risk score analysis. 26

SUPPLEMENTARY LIFELINES GROUP AUTHOR INFORMATION

UMCG Genetics Lifelines Initiative (UGLI) group author: LifeLines Cohort Study

Raul Aguirre-Gamboa (1), Patrick Deelen (1), Lude Franke (1), Jan A Kuivenhoven (2), Esteban A Lopera Maya (1), Ilja M Nolte (3), Serena Sanna (1), Harold Snieder (3), Morris A Swertz (1), Peter M. Visscher (3,4), Judith M Vonk (3), Cisca Wijmenga (1), Naomi Wray (4)

(1) Department of Genetics, University of Groningen, University Medical Center Groningen, The Netherlands

(2) Department of Pediatrics, University of Groningen, University Medical Center Groningen, The Netherlands

(3) Department of Epidemiology, University of Groningen, University Medical Center Groningen, The Netherlands

(4) Institute for Molecular Bioscience, The University of Queensland, Brisbane, Queensland, Australia.

SUPPLEMENTARY METHODS

1.1. Cogstate Test Battery

Detection Task. This is a reaction time task designed to assess psychomotor functioning and processing speed (Kuiper et al., 2017). In this task, participants attend to a card in the centre of the screen and respond to the question “Has the card turned face up” with “Yes” as soon as the card faces up. The task ends after 35 correct trials. The primary outcome is reaction time (ms) normalised using log10 transformation.

Identification Task. This is a reaction time task designed to measure visual attention (Kuiper et al., 2017). In this task, participants attend to a card in the centre of the screen and respond to the question “Is the card red?” with “Yes” or “No”. The task ends after 30 correct trials. The primary outcome is reaction time (ms) normalized using log10 transformation.

One-back Task. This task is designed to measure of attention and working memory (Kuiper et al., 2017). In this task, participants attend to a card in the centre of the screen and respond to the question “Is this card the same as that on the immediately previous trial?” with “Yes” or “No”. The task ends after 30 correct trials. The primary outcome is proportion of correct answers, normalized using arcsine transformation.

One Card Learning Task. This task is designed to measure visual learning and memory (Kuiper et al., 2017). In this task, participants attend to a card in the centre of the screen and respond to the question “Have you seen this card before in this task?” with “Yes” or “No”. The task ends after 42 trials. The primary outcome is proportion of correct answers normalized using arcsine transformation.

1.2. Lifelines Genetics Data

CytoSNP. In total, ~17,000 participants were genotyped in different batches using Illumina HumanCytoSNP-12v2.0 (n~16,500) and HumanCytoSNP-12v2.1 (n~500). Only probes present on both platforms were included. Genotyping was done using OptiCall and calls were refined using Beagle. CytoSNP originally used Genome Build 36, the probes were remapped to Genome Build 37 using SHRiMP2, with all probes are mapped on the forward strand. In total, 264,922 variants were present on both versions of the used HumanCytoSNP. Quality controls included excluding individuals with: (1) gender mismatches, (2) minimal or excessive heterozygosity, (3) duplicate sample identification, (4) missingness (call-rate < 95%), (5) non-Caucasian (determined by self-report in Lifelines phenotype database, Outlier [IBS] analysis, population stratification using Eigenstrat), (6) cryptic relationships (if a pair of samples were indicated as first-degree relatives using genetic similarity, the sample with the best genotyping quality was included). This resulted in 15,422 participants being available. Variants with minor allele frequency (MAF) of < 1%, call rate < 95%, or evidence for violations of Hardy-Weinberg Equilibrium ($p < 0.001$) were removed. Phasing was done using SHAPEIT2 and imputation using IMPUTE2 (combined reference panel of both genomes Genome of the Netherlands release 5 and 1000 Genomes phase1 v3 was used). For further details, please see: http://wiki-lifelines.web.rug.nl/doku.php?id=gwas.

GSA [UGLI cohort]. In total, 38,030 participants were genotyped in 31 batches using the Infinium Global Screening Array® (GSA) MultiEthnic Disease version 1.0. In total, 691,072 variants were genotyped, of which 571,420 markers met quality control steps. Quality controls included excluding individuals with: (1) gender mismatches, (2) heterozygosity (>4 standard deviations from mean), (3) duplicate samples, (4) missingness (two-step process: first removed individuals > 20% missingness and then > 1% missingness). Participants were of Caucasian-ancestry, although PCA analysis detected 35 participants who were non-European. Variants which were monomorphic (MAF = 0), call rate < 1%, HWE ($p \leq 1x10^{-6}$) were removed. A final set of 36,339 participants and 571,420 variants on autosomal and X chromosomes passed quality steps described above and were used for genetic imputation. Genetic imputation was done through the Sanger imputation service using the Haplotype Reference Consortium (http://www.haplotype-reference-consortium.org) panel. Following format instructions from the Sanger webpage (https://www.sanger.ac.uk/science/tools/sanger-imputation-service), 152 tri-allelic variants and 1608 insertions/deletions were removed. To facilitate researchers, Lifelines provide a list of non-European participants and a list of poorly imputed SNPs. For further details, please see: http://wiki-lifelines.web.rug.nl/lib/exe/fetch.php?media=qc_report_ugli_r1.pdf and http://wiki-lifelines.web.rug.nl/doku.php?id=ugli.

Affymetrix [UGLI2 cohort]. In total, 29,166 participants were genotyped in 12 batches using the FinnGen Thermo Fisher Axiom® Custom array. Genotyping was done in Human Genome Build hg38. Quality controls included excluding individuals with: (1) sample mix-ups (using gender mismatches and pedigree concordance), (2) heterozygosity (> 4 SD from the mean), (3) duplicate sample, (4) missingness (two-step process: first removed individuals with > 20%

missingness and then > 3% missingness). A genetic relationship matrix was created for the 1000G cohort (without the admixed AMR population samples) (https://www.internationalgenome.org/) and used for principle-component-analysis (PCA) of up to 20 principal components to generate PC-loadings that were projected onto the UGLI2 cohort. The PC analysis of all 1000G superpopulations identified 142 non-Europeans (>4 SDs from centroid 1000G European population for first five PCs), and PC analysis of only 1000G European population identified 161 genetic outliers (>4 SDs from centroid of all UGLI2 samples for first two PCs). In this study, we removed non-European and genetic outliers from the dataset. Variants with MAF < 0.02%, call rate < 1%, HWE in all samples ($p<1x10^{-10}$), HWE in unrelated samples ($p<1x10^{-6}$; defined as no 1st or 2nd degree relations) were excluded. There were no SNPs with > 1% Mendelian errors across all parent-offspring pairs. Prior to imputation, genetic markers were lifted over to Genome Build GRCh37 and aligned with Haplotype Reference Consortium (HRC) v1.1 (http://www.haplotype-referenceconsortium.org/site). A final set of 28,250 samples and 462,731 markers on autosomal and X chromosomes passing quality check steps above were used for genetic imputation using the Sanger imputation service using HRC panel. For further details, please see: http://wiki-lifelines.web.rug.nl/lib/exe/fetch.php?media=qc_report_ugli2_release_1_-v1.pdf and http://wiki-lifelines.web.rug.nl/doku.php?id=ugli.

Principal Components. Lifelines provides genetic PCs for each chip separately, and PCs created using combined data (using all individuals). For the GRAMMAR method, PCs calculated on each chip separately were used. For unadjusted analysis and analysis removing related individuals, combined PCs were used.

1.3. Details of GWAS and Instruments used to create GRS in Lifelines

Said et al. (2022). Meta analysis of two GWAS:

CHARGE Consortium (Ligthart et al., 2018) Circulating CRP was natural log transformed. Individuals were excluded from analyses if they had an auto-immune disease, were taking immune-modulating agents (if information was available) or had CRP ≥ 4 SD from the mean.

UK Biobank Circulating CRP was natural log transformed, and individuals with extreme values (± 4 SD) from the mean were excluded. Individuals taking immune modulating drugs, who had auto-immune related conditions (1.8% sample), were removed.

Ahluwalia et al. (2021) Circulating IL-6 was natural log transformed. Only population-based samples or healthy controls from case-control studies were included.

Sarwar et al. (2012) Circulating IL-6 was natural log transformed. This instrument is composed of a single SNP (rs2228145) in the *IL6R* gene. The minor allele of this SNP (358A1a) is associated with increased sIL6R levels and decreased CRP levels[4,5]. Cell based experiments show that the minor allele decreases classical signalling [4]. Therefore, the effect

of this SNP can be seen as a proxy for IL-6 activity. For more information on the biological function of this SNP, see [4,6].

Borges et al. (2020) Details not available.

Rosa et al. (2019) This SNP list was based on the Sun et al. (2018) sIL6R GWAS. The GWAS includes participants in good health. Individuals were excluded if they had a history of major disease (such as myocardial infarction, stroke, cancer, HIV, and hepatitis B or C) or and had a recent illness or infection. For more information, see[8]. Quality controls included exclusions for sex mismatches, low call rates, duplicate sample, extreme heterozygosity, and non-European descent.

1.4. Adjustment for relatedness in genetic analyses

To adjust for relatedness within each chip, two approaches were taken. First, we applied the GRAMMAR method *(*primary analysis)[9]: on each chip, we performed restricted estimated maximum likelihood predicting each outcome with the sparse genetic relationship matrix (GRM) and top 10 genetic principal components (PCs) as predictors. This was done using --*reml-pred-rand* in GCTA[10]. We extracted the residuals from each model and merged the data across chips (standardized GRS, residuals, age, sex). We then performed linear regressions to predict the outcome residuals using standardized GRS, age, sex, and chip (to adjust for potential differences between chips). Second, we re-ran analysis removing close relatives (secondary analysis) using *king-cutoff* [11] in Plink v2.0 [12,13] to identify relatives using conventional cut-offs for kinship coefficients (up to first-degree: 0.177; up to second-degree: 0.088; up to third-degree: 0.044).

1.5. Creating Genetic Relationship Matrices (GRM)

Using genotype (non-imputed) data, we created three GRM's (one for each chip). SNPs were used to create the GRM if they met the following criteria: MAF (0.01), call rate (0.95), HWE (1e-6), not multi-allelic. Independent SNPs were selected using --*indep-pairwise* [50 10 0.1] in Plink v1.9. Following this, a sparse GRM was created by replacing non-diagonal values <0.125 to 0 using --*make-bK* in GCTA[10]. A sparse matrix was created so that inclusion of the GRM will only adjust for recent relatedness.

SUPPLEMENTARY RESULTS

2.1. GRS analysis

Primary analyses were run adjusted for close relatedness within chips using a genetic relationship matrix (GRAMMAR method). Secondary analyses included (1) re-running the

analysis unadjusted for relatedness within chips and (2) re-running the analysis removing close relatives within chips (up to first-degree, up to second-degree, up to third-degree). Conclusions are consistent across analyses.

2.2. GRSs associations with exposures and potential confounders

Linear regression models checked whether CRP GRS's predicted circulating levels of CRP in 23,607 Lifelines participants who had both genetic and CRP data available. The GRAMMAR method was used. All instruments had *F*-statistics >10 (158 for *cis* GRS, 1045 for genome-wide GRS), indicating adequate instrument strength[14,15]. *Cis* and genome-wide instruments explained 0.7% and 4.2% variance in CRP levels, respectively. The amount of variance explained by CRP GRS's in circulating CRP is consistent with our previous results in the ALSPAC cohort[16].

Linear regression models examined whether GRS's were associated with potential confounders. For primary instruments, there was evidence that the CRP *cis* instrument was associated with smoking (p=0.035) and weak evidence that the sIL6R instrument was associated with lower education attainment (p=0.061). For secondary instruments, there was evidence that the CRP genome-wide instrument was associated with BMI (p<0.001), smoking (p=0.0003) and lower education attainment (p=0.0004). Evidence for other GRS's was weak (Supplementary Table 3). Stronger evidence of associations between genome-wide instruments and potential confounders (consistent to what was reported in the previous ALSPAC study) highlights that *cis* variants may provide more valid instruments for MR [16].

SUPPLEMENTARY DISCUSSION

3.1. Possible factors contributing to mixed MR results of CRP-depression

Four factors which may contribute are CRP SNP selection, how depression was measured, statistical power, and selection bias. Regarding SNP selection, all previous studies (including the current study) used *cis* CRP SNPs; and previous studies included the same four *cis* SNPs (rs1205, rs1130864, rs3093077, rs3091244) as either a primary or secondary instrument. Thus, it is unlikely that SNP selection is driving discrepancy. Depression has been characterized differently across studies. This includes self-reported probable lifetime depression[17], hospitalization or death with depression[18], depression questionnaires/interviews assessed using PHQ-9 and HADS-D (coded continuously and categorically) [19,20] or MINI (current study), or a mixture of the above[21]. Different measures assess different symptoms of depression (e.g., the PHQ-9 includes somatic symptoms which are not included in measures such as HADS-D[20]). Therefore, if CRP differentially affects specific depression symptoms, this may contribute to mixed results observed. Selection bias

should also be considered. For example, the study by Ye et al. (2021) which reported higher CRP to be associated with decreased risk of depression only included a subset of UK Biobank participants who responded to a mental health survey at follow-up, and consequently may be affected by selection bias.

SUPPLEMENTARY TABLES

Table 1. Access to GWAS Data

GWAS Full Summary Statistics/Instruments	Access	URL *(if available online)* or author contact details
Said et al. (2022)	Requested from authors	https://www.ebi.ac.uk/gwas/studies/GCST90029070
Ahluwalia et al. (2021)	Requested from authors	Corresponding authors:<br>Tarunveer Ahluwalia<br>(email: tarun.veer.singh.ahluwalia@regionh.dk)<br>Behrooz Alizadeh<br>(email: b.z.alizadeh@umcg.nl)
Sarwar et al. (2012)	Taken from OSF	osf.io/apme9/
Borges et al. (2020)	IEU Open GWAS Project	gwas.mrcieu.ac.uk/datasets/met-d-GlycA/
Rosa et al. (2019)	Available in paper supplementary	www.nature.com/articles/s41525-019-0097-4#Sec30

Table 2. Details of GWAS used to create GRS

Phenotype	GWAS/ Instrument	Population	Cohort/ studies(*s*)	Covariates	Ages	N	Includes Lifelines (approximate % sample if applicable)	Ref
**CRP**	Said et al. (2022)	European ancestry	Meta-analysis of GWAS from CHARGE consortium (n=148,164) and UK Biobank (n=427,367).	CHARGE adjusted for age, sex, population structure, accounting for relatedness, if relevant.	CHARGE consortium cohorts range from *M* age of 9.9 to 86.6 years<br><br>UK Biobank age (M=56.5, SD=8.1)	575,531	Yes, Lifelines Cohort is in CHARGE GWAS; n ≤ **2.2%.**	1
**IL-6/IL-6R**	Ahluwalia et al. (2021)	European ancestry	26 cohorts	Adjusted for age, sex, population substructure (through study-specific principal components) and/or study-specific site, when necessary.	Cohorts range from *M* age of 9.9 to 86.6 years	52,654	No	2
	Sarwar et al. (2012) Instrument	European ancestry (≥ 90%)	16 studies	Unknown	Unknown	27,185	No	3
**GlycA**	Borges et al. (2020)	European	UK Biobank	Unknown	Unknown	115,078	No	N/A
**sIL6R**	Rosa et al. (2019) Instrument from Sun et al., (2018) GWAS on sIL6R.	European ancestry	INTERVAL study (UK)	Adjusted for sex, age, duration between blood draw and processing, first 3 ancestry principal components.	Cohorts *M* age is 44 years (*SD* = 14)	3,301	No	7,8

Table 3. Standardised GRS on potential confounders (GRAMMAR adjusted)

Standardised GRS	Outcome	Beta	p-value	LCI	UCI	N
CRP (said-cis)	BMI	-0.00399	0.6267	-0.02005	0.01208	58680
CRP (said-cis)	Current smoke status	0.00272	0.0347	0.00020	0.00524	57935
CRP (said-cis)	Education attainment	0.00163	0.3885	-0.00207	0.00533	58094
GlycA (borges)	BMI	0.00541	0.5094	-0.01066	0.02147	58680
GlycA (borges)	Current smoke status	0.00140	0.2777	-0.00113	0.00392	57935
GlycA (borges)	Education attainment	0.00068	0.7169	-0.00302	0.00439	58094
IL-6R (ahluwalia-cis)	BMI	-0.00305	0.7102	-0.01911	0.01302	58680
IL-6R (ahluwalia-cis)	Current smoke status	0.00156	0.2271	-0.00097	0.00408	57935
IL-6R (ahluwalia-cis)	Education attainment	0.00199	0.2928	-0.00172	0.00569	58094
sIL6R (rosa)	BMI	0.00669	0.4141	-0.00937	0.02276	58680
sIL6R (rosa)	Current smoke status	0.00159	0.2173	-0.00094	0.00411	57935
sIL6R (rosa)	Education attainment	0.00354	0.0608	-0.00016	0.00724	58094
IL-6R (sarwar)	BMI	0.00172	0.8339	-0.01435	0.01779	58680
IL-6R (sarwar)	Current smoke status	0.00124	0.3371	-0.00129	0.00376	57935
IL-6R (sarwar)	Education attainment	0.00192	0.3103	-0.00179	0.00562	58094
CRP (said-genome-wide)	BMI	0.04719	<0.0001	0.03113	0.06324	58680
CRP (said-genome-wide)	Current smoke status	0.00465	0.0003	0.00213	0.00717	57935
CRP (said-genome-wide)	Education attainment	0.00671	0.0004	0.00301	0.01041	58094
IL-6 (ahluwalia-genome-wide)	BMI	-0.00408	0.6194	-0.02016	0.01201	58680
IL-6 (ahluwalia-genome-wide)	Current smoke status	0.00125	0.3325	-0.00128	0.00378	57935
IL-6 (ahluwalia-genome-wide)	Education attainment	0.00211	0.2655	-0.00160	0.00581	58094

Table 4. Standardised GRS on standardised outcomes (GRAMMAR adjusted).

Standardised GRS	Outcome	Beta	p-value	LCI	UCI	N
CRP (said-cis)	Identify Task – reaction time	-0.00251	0.502	-0.00986	0.00483	35729
CRP (said-cis)	Detect Task – reaction time	0.00389	0.355	-0.00435	0.01213	35300
CRP (said-cis)	OCL Task - accuracy	-0.00566	0.153	-0.01341	0.00210	36783
CRP (said-cis)	One-back Task - accuracy	-0.00646	0.159	-0.01547	0.00254	36349
CRP (said-cis)	RFFT	0.00460	0.217	-0.00271	0.01191	36563
CRP (said-cis)	PANAS – negative	0.00646	0.035	0.00046	0.01247	57946
CRP (said-cis)	PANAS - positive	0.00405	0.192	-0.00203	0.01014	57946
CRP (said-cis)	Mini depression	0.00079	0.217	-0.00046	0.00204	56860
CRP (said-cis)	Mini anxiety	0.00198	0.037	0.00012	0.00384	57047
CRP (said-cis)	Mini MDD	0.00037	0.486	-0.00067	0.00140	57047
CRP (said-cis)	Mini GAD	0.00101	0.164	-0.00041	0.00243	57047
GlycA (borges)	Identify Task – reaction time	-0.00002	0.996	-0.00737	0.00733	35729
GlycA (borges)	Detect Task – reaction time	0.00093	0.826	-0.00732	0.00918	35300
GlycA (borges)	OCL Task - accuracy	0.00232	0.558	-0.00544	0.01007	36783
GlycA (borges)	One-back Task - accuracy	0.00110	0.810	-0.00791	0.01011	36349
GlycA (borges)	RFFT	-0.00541	0.148	-0.01273	0.00191	36563
GlycA (borges)	PANAS – negative	0.00603	0.049	0.00002	0.01203	57946
GlycA (borges)	PANAS - positive	-0.00185	0.552	-0.00794	0.00424	57946
GlycA (borges)	Mini depression	0.00087	0.172	-0.00038	0.00212	56860
GlycA (borges)	Mini anxiety	0.00021	0.821	-0.00164	0.00207	57047
GlycA (borges)	Mini MDD	0.00110	0.036	0.00007	0.00213	57047
GlycA (borges)	Mini GAD	0.00053	0.465	-0.00089	0.00195	57047
IL-6R (ahluwalia-cis)	Identify Task – reaction time	-0.00504	0.179	-0.01239	0.00232	35729
IL-6R (ahluwalia-cis)	Detect Task – reaction time	-0.00144	0.733	-0.00970	0.00682	35300
IL-6R (ahluwalia-cis)	OCL Task - accuracy	-0.00394	0.320	-0.01170	0.00383	36783
IL-6R (ahluwalia-cis)	One-back Task - accuracy	0.00171	0.710	-0.00730	0.01072	36349
IL-6R (ahluwalia-cis)	RFFT	-0.00395	0.291	-0.01127	0.00338	36563
IL-6R (ahluwalia-cis)	PANAS – negative	0.00348	0.257	-0.00253	0.00949	57946
IL-6R (ahluwalia-cis)	PANAS - positive	-0.00099	0.750	-0.00708	0.00510	57946
IL-6R (ahluwalia-cis)	Mini depression	-0.00024	0.708	-0.00149	0.00101	56860

IL-6R (ahluwalia-cis)	Mini anxiety	-0.00022	0.819	-0.00208	0.00164	57047
IL-6R (ahluwalia-cis)	Mini MDD	-0.00002	0.964	-0.00106	0.00101	57047
IL-6R (ahluwalia-cis)	Mini GAD	0.00013	0.857	-0.00129	0.00155	57047
sIL6R (rosa)	Identify Task – reaction time	-0.00423	0.259	-0.01158	0.00312	35729
sIL6R (rosa)	Detect Task – reaction time	-0.00080	0.849	-0.00905	0.00746	35300
sIL6R (rosa)	OCL Task - accuracy	-0.00936	0.018	-0.01712	-0.00161	36783
sIL6R (rosa)	One-back Task - accuracy	-0.00442	0.336	-0.01343	0.00459	36349
sIL6R (rosa)	RFFT	-0.00063	0.867	-0.00793	0.00668	36563
sIL6R (rosa)	PANAS – negative	0.00459	0.134	-0.00142	0.01060	57946
sIL6R (rosa)	PANAS - positive	-0.00228	0.462	-0.00837	0.00381	57946
sIL6R (rosa)	Mini depression	-0.00057	0.369	-0.00182	0.00068	56860
sIL6R (rosa)	Mini anxiety	-0.00005	0.960	-0.00191	0.00181	57047
sIL6R (rosa)	Mini MDD	-0.00009	0.863	-0.00112	0.00094	57047
sIL6R (rosa)	Mini GAD	0.00009	0.901	-0.00133	0.00151	57047
IL-6R (sarwar)	Identify Task – reaction time	-0.00434	0.246	-0.01168	0.00300	35729
IL-6R (sarwar)	Detect Task – reaction time	-0.00183	0.663	-0.01007	0.00641	35300
IL-6R (sarwar)	OCL Task - accuracy	-0.00588	0.137	-0.01363	0.00187	36783
IL-6R (sarwar)	One-back Task - accuracy	0.00117	0.798	-0.00782	0.01017	36349
IL-6R (sarwar)	RFFT	-0.00047	0.899	-0.00779	0.00684	36563
IL-6R (sarwar)	PANAS – negative	0.00373	0.223	-0.00227	0.00974	57946
IL-6R (sarwar)	PANAS - positive	-0.00443	0.154	-0.01052	0.00166	57946
IL-6R (sarwar)	Mini depression	-0.00007	0.915	-0.00132	0.00118	56860
IL-6R (sarwar)	Mini anxiety	0.00029	0.762	-0.00157	0.00215	57047
IL-6R (sarwar)	Mini MDD	0.00003	0.948	-0.00100	0.00107	57047
IL-6R (sarwar)	Mini GAD	0.00034	0.637	-0.00108	0.00176	57047
CRP (said-genome-wide)	Identify Task – reaction time	-0.00421	0.260	-0.01155	0.00312	35729
CRP (said-genome-wide)	Detect Task – reaction time	0.00185	0.659	-0.00637	0.01007	35300
CRP (said-genome-wide)	OCL Task - accuracy	0.00360	0.362	-0.00414	0.01133	36783
CRP (said-genome-wide)	One-back Task - accuracy	0.00429	0.349	-0.00468	0.01327	36349
CRP (said-genome-wide)	RFFT	0.00290	0.437	-0.00442	0.01022	36563
CRP (said-genome-wide)	PANAS – negative	-0.00221	0.471	-0.00821	0.00380	57946
CRP (said-genome-wide)	PANAS - positive	-0.00058	0.852	-0.00666	0.00551	57946

CRP (said-genome-wide)	Mini depression	0.00060	0.350	-0.00065	0.00184	56860
CRP (said-genome-wide)	Mini anxiety	0.00215	0.023	0.00029	0.00401	57047
CRP (said-genome-wide)	Mini MDD	0.00031	0.561	-0.00072	0.00134	57047
CRP (said-genome-wide)	Mini GAD	0.00078	0.279	-0.00063	0.00220	57047
IL-6 (ahluwalia-genome-wide)	Identify Task – reaction time	-0.00463	0.217	-0.01199	0.00272	35729
IL-6 (ahluwalia-genome-wide)	Detect Task – reaction time	-0.00143	0.735	-0.00969	0.00684	35300
IL-6 (ahluwalia-genome-wide)	OCL Task - accuracy	-0.00390	0.325	-0.01166	0.00387	36783
IL-6 (ahluwalia-genome-wide)	One-back Task - accuracy	0.00157	0.733	-0.00744	0.01059	36349
IL-6 (ahluwalia-genome-wide)	RFFT	-0.00451	0.228	-0.01184	0.00283	36563
IL-6 (ahluwalia-genome-wide)	PANAS – negative	0.00432	0.159	-0.00169	0.01033	57946
IL-6 (ahluwalia-genome-wide)	PANAS - positive	0.00009	0.976	-0.00600	0.00619	57946
IL-6 (ahluwalia-genome-wide)	Mini depression	-0.00011	0.865	-0.00136	0.00114	56860
IL-6 (ahluwalia-genome-wide)	Mini anxiety	0.00015	0.875	-0.00171	0.00201	57047
IL-6 (ahluwalia-genome-wide)	Mini MDD	0.00002	0.969	-0.00101	0.00105	57047
IL-6 (ahluwalia-genome-wide)	Mini GAD	0.00029	0.693	-0.00113	0.00171	57047

Table 5. Standardised GRS on binary outcomes (unadjusted analysis)

Standardised GRS	Outcome	OR	p-value	LCI	UCI	N
CRP (said-cis)	Mini depression	1.034	0.185	0.984	1.085	56860
CRP (said-cis)	Mini anxiety	1.035	0.034	1.003	1.068	57047
CRP (said-cis)	Mini MDD	1.022	0.487	0.961	1.087	57047
CRP (said-cis)	Mini GAD	1.030	0.175	0.987	1.075	57047
GlycA (borges)	Mini depression	1.031	0.224	0.982	1.083	56860
GlycA (borges)	Mini anxiety	1.003	0.876	0.971	1.035	57047
GlycA (borges)	Mini MDD	1.068	0.037	1.004	1.137	57047
GlycA (borges)	Mini GAD	1.016	0.481	0.973	1.060	57047
IL-6R (ahluwalia)	Mini depression	0.990	0.693	0.942	1.040	56860
IL-6R (ahluwalia)	Mini anxiety	0.994	0.731	0.963	1.027	57047
IL-6R (ahluwalia)	Mini MDD	0.999	0.979	0.939	1.063	57047
IL-6R (ahluwalia)	Mini GAD	1.001	0.972	0.958	1.045	57047
sIL6R (rosa)	Mini depression	0.976	0.325	0.929	1.025	56860
sIL6R (rosa)	Mini anxiety	0.997	0.854	0.966	1.029	57047
sIL6R (rosa)	Mini MDD	0.995	0.871	0.935	1.059	57047
sIL6R (rosa)	Mini GAD	0.999	0.963	0.957	1.043	57047
IL-6R (sarwar)	Mini depression	0.996	0.887	0.948	1.047	56860
IL-6R (sarwar)	Mini anxiety	1.003	0.861	0.971	1.035	57047
IL-6R (sarwar)	Mini MDD	1.003	0.930	0.942	1.067	57047
IL-6R (sarwar)	Mini GAD	1.007	0.752	0.964	1.051	57047
CRP (said-genome-wide)	Mini depression	1.024	0.346	0.975	1.076	56860
CRP (said-genome-wide)	Mini anxiety	1.040	0.016	1.007	1.074	57047
CRP (said-genome-wide)	Mini MDD	1.016	0.627	0.954	1.081	57047
CRP (said-genome-wide)	Mini GAD	1.025	0.254	0.982	1.071	57047
IL-6 (ahluwalia-genome-wide)	Mini depression	0.995	0.832	0.947	1.045	56860
IL-6 (ahluwalia-genome-wide)	Mini anxiety	0.998	0.892	0.966	1.030	57047
IL-6 (ahluwalia-genome-wide)	Mini MDD	1.004	0.888	0.944	1.069	57047
IL-6 (ahluwalia-genome-wide)	Mini GAD	1.003	0.897	0.960	1.047	57047

Table 6. Standardised GRS on continuous outcomes (unadjusted analysis)

Standardised GRS	Standardised Outcomes	Beta	p-value	LCI	UCI	N
CRP (said-cis)	Identify Task – reaction time	-0.00321	0.492	-0.01235	0.00594	35729
CRP (said-cis)	Detect Task – reaction time	0.00437	0.359	-0.00497	0.01372	35300
CRP (said-cis)	OCL Task - accuracy	-0.00740	0.146	-0.01737	0.00257	36783
CRP (said-cis)	One-back Task - accuracy	-0.00627	0.219	-0.01626	0.00372	36349
CRP (said-cis)	RFFT	0.00514	0.299	-0.00455	0.01483	36563
CRP (said-cis)	PANAS – negative	0.00805	0.046	0.00016	0.01594	57946
CRP (said-cis)	PANAS - positive	0.00535	0.191	-0.00266	0.01337	57946
GlycA (borges)	Identify Task – reaction time	-0.00069	0.882	-0.00984	0.00846	35729
GlycA (borges)	Detect Task – reaction time	0.00189	0.692	-0.00747	0.01125	35300
GlycA (borges)	OCL Task - accuracy	0.00307	0.547	-0.00691	0.01304	36783
GlycA (borges)	One-back Task - accuracy	0.00083	0.870	-0.00916	0.01083	36349
GlycA (borges)	RFFT	-0.00652	0.188	-0.01623	0.00319	36563
GlycA (borges)	PANAS – negative	0.00916	0.023	0.00126	0.01706	57946
GlycA (borges)	PANAS - positive	-0.00307	0.453	-0.01109	0.00495	57946
IL-6R (ahluwalia-cis)	Identify Task – reaction time	-0.00574	0.219	-0.01489	0.00341	35729
IL-6R (ahluwalia-cis)	Detect Task – reaction time	-0.00084	0.860	-0.01021	0.00853	35300
IL-6R (ahluwalia-cis)	OCL Task - accuracy	-0.00504	0.323	-0.01502	0.00495	36783
IL-6R (ahluwalia-cis)	One-back Task - accuracy	0.00143	0.780	-0.00857	0.01142	36349
IL-6R (ahluwalia-cis)	RFFT	-0.00609	0.219	-0.01580	0.00362	36563
IL-6R (ahluwalia-cis)	PANAS – negative	0.00402	0.318	-0.00387	0.01192	57946
IL-6R (ahluwalia-cis)	PANAS - positive	-0.00156	0.704	-0.00957	0.00646	57946
sIL6R (rosa)	Identify Task – reaction time	-0.00482	0.301	-0.01397	0.00432	35729
sIL6R (rosa)	Detect Task – reaction time	0.00002	0.996	-0.00933	0.00938	35300
sIL6R (rosa)	OCL Task - accuracy	-0.01197	0.019	-0.02195	-0.00200	36783
sIL6R (rosa)	One-back Task - accuracy	-0.00545	0.285	-0.01545	0.00454	36349
sIL6R (rosa)	RFFT	-0.00128	0.795	-0.01097	0.00840	36563
sIL6R (rosa)	PANAS – negative	0.00567	0.159	-0.00223	0.01357	57946
sIL6R (rosa)	PANAS - positive	-0.00293	0.474	-0.01095	0.00509	57946
IL-6R (sarwar)	Identify Task – reaction time	-0.00497	0.286	-0.01410	0.00417	35729
IL-6R (sarwar)	Detect Task – reaction time	-0.00120	0.802	-0.01054	0.00815	35300

IL-6R (sarwar)	OCL Task - accuracy	-0.00772	0.129	-0.01769	0.00224	36783
IL-6R (sarwar)	One-back Task - accuracy	0.00065	0.899	-0.00933	0.01062	36349
IL-6R (sarwar)	RFFT	-0.00186	0.706	-0.01156	0.00783	36563
IL-6R (sarwar)	PANAS – negative	0.00441	0.274	-0.00349	0.01230	57946
IL-6R (sarwar)	PANAS - positive	-0.00602	0.141	-0.01404	0.00200	57946
CRP (said-genome-wide)	Identify Task – reaction time	-0.00477	0.305	-0.01389	0.00435	35729
CRP (said-genome-wide)	Detect Task – reaction time	0.00253	0.595	-0.00679	0.01185	35300
CRP (said-genome-wide)	OCL Task - accuracy	0.00518	0.307	-0.00477	0.01512	36783
CRP (said-genome-wide)	One-back Task - accuracy	0.00607	0.232	-0.00388	0.01603	36349
CRP (said-genome-wide)	RFFT	0.00498	0.314	-0.00471	0.01468	36563
CRP (said-genome-wide)	PANAS – negative	-0.00411	0.308	-0.01199	0.00378	57946
CRP (said-genome-wide)	PANAS - positive	-0.00006	0.989	-0.00807	0.00795	57946
IL-6 (ahluwalia-genome-wide)	Identify Task – reaction time	-0.00580	0.215	-0.01498	0.00338	35729
IL-6 (ahluwalia-genome-wide)	Detect Task – reaction time	-0.00149	0.756	-0.01089	0.00791	35300
IL-6 (ahluwalia-genome-wide)	OCL Task - accuracy	-0.00400	0.434	-0.01401	0.00602	36783
IL-6 (ahluwalia-genome-wide)	One-back Task - accuracy	0.00133	0.794	-0.00870	0.01136	36349
IL-6 (ahluwalia-genome-wide)	RFFT	-0.00604	0.225	-0.01578	0.00371	36563
IL-6 (ahluwalia-genome-wide)	PANAS – negative	0.00423	0.295	-0.00369	0.01216	57946
IL-6 (ahluwalia-genome-wide)	PANAS - positive	0.00041	0.921	-0.00764	0.00846	57946

Table 7. Standardised GRS on binary outcomes (1st degree relatives removed)

Standardised GRS	Outcome	OR	p-value	LCI	UCI	N
CRP (said-cis)	Mini depression	1.048	0.084	0.994	1.104	49183
CRP (said-cis)	Mini anxiety	1.035	0.048	1.000	1.071	49348
CRP (said-cis)	Mini MDD	1.041	0.238	0.974	1.112	49348
CRP (said-cis)	Mini GAD	1.036	0.137	0.989	1.085	49348
GlycA (borges)	Mini depression	1.046	0.096	0.992	1.103	49183
GlycA (borges)	Mini anxiety	1.016	0.364	0.982	1.051	49348
GlycA (borges)	Mini MDD	1.083	0.019	1.013	1.158	49348
GlycA (borges)	Mini GAD	1.046	0.056	0.999	1.096	49348
IL-6R (ahluwalia)	Mini depression	1.005	0.858	0.953	1.059	49183
IL-6R (ahluwalia)	Mini anxiety	1.001	0.973	0.967	1.035	49348
IL-6R (ahluwalia)	Mini MDD	1.001	0.974	0.936	1.070	49348
IL-6R (ahluwalia)	Mini GAD	1.006	0.816	0.960	1.053	49348
sIL6R (rosa)	Mini depression	0.987	0.639	0.936	1.041	49183
sIL6R (rosa)	Mini anxiety	0.998	0.921	0.965	1.033	49348
sIL6R (rosa)	Mini MDD	0.997	0.929	0.932	1.066	49348
sIL6R (rosa)	Mini GAD	1.003	0.903	0.957	1.050	49348
IL-6R (sarwar)	Mini depression	1.008	0.769	0.956	1.063	49183
IL-6R (sarwar)	Mini anxiety	1.011	0.534	0.977	1.046	49348
IL-6R (sarwar)	Mini MDD	1.005	0.880	0.940	1.074	49348
IL-6R (sarwar)	Mini GAD	1.013	0.594	0.967	1.061	49348
CRP (said-genome-wide)	Mini depression	1.038	0.172	0.984	1.095	49183
CRP (said-genome-wide)	Mini anxiety	1.038	0.032	1.003	1.074	49348
CRP (said-genome-wide)	Mini MDD	1.027	0.429	0.961	1.098	49348
CRP (said-genome-wide)	Mini GAD	1.028	0.246	0.981	1.077	49348
IL-6 (ahluwalia-genome-wide)	Mini depression	1.010	0.701	0.958	1.066	49183
IL-6 (ahluwalia-genome-wide)	Mini anxiety	1.004	0.802	0.970	1.039	49348
IL-6 (ahluwalia-genome-wide)	Mini MDD	1.008	0.820	0.942	1.077	49348
IL-6 (ahluwalia-genome-wide)	Mini GAD	1.008	0.733	0.962	1.056	49348

Table 8. Standardised GRS on binary outcomes (2nd degree relatives removed)

Standardised GRS	Outcome	OR	p-value	LCI	UCI	N
CRP (said-cis)	Mini depression	1.051	0.064	0.997	1.109	48493
CRP (said-cis)	Mini anxiety	1.037	0.039	1.002	1.073	48656
CRP (said-cis)	Mini MDD	1.045	0.195	0.977	1.118	48656
CRP (said-cis)	Mini GAD	1.036	0.140	0.988	1.085	48656
GlycA (borges)	Mini depression	1.046	0.100	0.991	1.103	48493
GlycA (borges)	Mini anxiety	1.016	0.355	0.982	1.052	48656
GlycA (borges)	Mini MDD	1.085	0.018	1.014	1.160	48656
GlycA (borges)	Mini GAD	1.047	0.056	0.999	1.097	48656
IL-6R (ahluwalia)	Mini depression	1.001	0.985	0.948	1.055	48493
IL-6R (ahluwalia)	Mini anxiety	1.002	0.921	0.968	1.037	48656
IL-6R (ahluwalia)	Mini MDD	0.989	0.751	0.924	1.058	48656
IL-6R (ahluwalia)	Mini GAD	1.000	0.999	0.954	1.048	48656
sIL6R (rosa)	Mini depression	0.985	0.591	0.934	1.040	48493
sIL6R (rosa)	Mini anxiety	1.000	0.993	0.966	1.035	48656
sIL6R (rosa)	Mini MDD	0.991	0.790	0.926	1.060	48656
sIL6R (rosa)	Mini GAD	1.000	0.990	0.955	1.048	48656
IL-6R (sarwar)	Mini depression	1.004	0.885	0.952	1.059	48493
IL-6R (sarwar)	Mini anxiety	1.012	0.492	0.978	1.047	48656
IL-6R (sarwar)	Mini MDD	0.995	0.879	0.930	1.064	48656
IL-6R (sarwar)	Mini GAD	1.007	0.776	0.961	1.055	48656
CRP (said-genome-wide)	Mini depression	1.044	0.117	0.989	1.101	48493
CRP (said-genome-wide)	Mini anxiety	1.040	0.026	1.005	1.077	48656
CRP (said-genome-wide)	Mini MDD	1.032	0.357	0.965	1.105	48656
CRP (said-genome-wide)	Mini GAD	1.029	0.237	0.982	1.078	48656
IL-6 (ahluwalia-genome-wide)	Mini depression	1.006	0.836	0.953	1.061	48493
IL-6 (ahluwalia-genome-wide)	Mini anxiety	1.006	0.737	0.972	1.041	48656
IL-6 (ahluwalia-genome-wide)	Mini MDD	0.995	0.889	0.930	1.065	48656
IL-6 (ahluwalia-genome-wide)	Mini GAD	1.003	0.900	0.957	1.051	48656

Table 9. Standardised GRS on binary outcomes (3rd degree relatives removed)

Standardised GRS	Outcome	OR	p-value	LCI	UCI	N
CRP (said-cis)	Mini depression	1.047	0.094	0.992	1.105	47136
CRP (said-cis)	Mini anxiety	1.035	0.052	1.000	1.072	47295
CRP (said-cis)	Mini MDD	1.039	0.265	0.971	1.112	47295
CRP (said-cis)	Mini GAD	1.031	0.210	0.983	1.081	47295
GlycA (borges)	Mini depression	1.037	0.190	0.982	1.095	47136
GlycA (borges)	Mini anxiety	1.018	0.321	0.983	1.054	47295
GlycA (borges)	Mini MDD	1.066	0.067	0.996	1.141	47295
GlycA (borges)	Mini GAD	1.046	0.064	0.997	1.097	47295
IL-6R (ahluwalia)	Mini depression	1.001	0.975	0.948	1.057	47136
IL-6R (ahluwalia)	Mini anxiety	1.003	0.858	0.969	1.039	47295
IL-6R (ahluwalia)	Mini MDD	0.986	0.682	0.920	1.055	47295
IL-6R (ahluwalia)	Mini GAD	1.011	0.657	0.964	1.060	47295
sIL6R (rosa)	Mini depression	0.985	0.590	0.933	1.040	47136
sIL6R (rosa)	Mini anxiety	0.999	0.937	0.964	1.034	47295
sIL6R (rosa)	Mini MDD	0.983	0.615	0.917	1.052	47295
sIL6R (rosa)	Mini GAD	1.006	0.808	0.959	1.055	47295
IL-6R (sarwar)	Mini depression	1.001	0.965	0.948	1.057	47136
IL-6R (sarwar)	Mini anxiety	1.010	0.562	0.976	1.046	47295
IL-6R (sarwar)	Mini MDD	0.988	0.727	0.922	1.058	47295
IL-6R (sarwar)	Mini GAD	1.014	0.579	0.966	1.063	47295
CRP (said-genome-wide)	Mini depression	1.039	0.171	0.984	1.097	47136
CRP (said-genome-wide)	Mini anxiety	1.040	0.027	1.004	1.078	47295
CRP (said-genome-wide)	Mini MDD	1.027	0.451	0.959	1.100	47295
CRP (said-genome-wide)	Mini GAD	1.023	0.359	0.975	1.073	47295
IL-6 (ahluwalia-genome-wide)	Mini depression	1.006	0.817	0.953	1.063	47136
IL-6 (ahluwalia-genome-wide)	Mini anxiety	1.008	0.674	0.973	1.044	47295
IL-6 (ahluwalia-genome-wide)	Mini MDD	0.992	0.824	0.926	1.063	47295
IL-6 (ahluwalia-genome-wide)	Mini GAD	1.014	0.560	0.967	1.064	47295

Table 10. Standardised GRS on continuous outcomes (1st degree relatives removed)

Standardised GRS	Standardised Outcomes	Beta	p-value	LCI	UCI	N
CRP (said-cis)	Identify Task – reaction time	-0.004049836	0.418	-0.013858126	0.005758454	31248
CRP (said-cis)	Detect Task – reaction time	0.007610043	0.137	-0.00240993	0.017630016	30872
CRP (said-cis)	OCL Task - accuracy	-0.007719335	0.156	-0.018391409	0.002952738	32182
CRP (said-cis)	One-back Task - accuracy	-0.003619921	0.506	-0.014281494	0.007041651	31805
CRP (said-cis)	RFFT	0.005215785	0.321	-0.005090252	0.015521822	32250
CRP (said-cis)	PANAS – negative	0.009218252	0.033	0.000758222	0.017678281	50275
CRP (said-cis)	PANAS - positive	0.005766673	0.189	-0.002841032	0.014374379	50275
GlycA (borges)	Identify Task – reaction time	0.002430414	0.626	-0.007341182	0.012202009	31248
GlycA (borges)	Detect Task – reaction time	0.004379256	0.390	-0.005610349	0.014368861	30872
GlycA (borges)	OCL Task - accuracy	0.003578283	0.510	-0.007055413	0.014211979	32182
GlycA (borges)	One-back Task - accuracy	-0.000115487	0.983	-0.010745161	0.010514187	31805
GlycA (borges)	RFFT	-0.005756899	0.274	-0.016072422	0.004558624	32250
GlycA (borges)	PANAS – negative	0.011174486	0.010	0.0027246	0.019624373	50275
GlycA (borges)	PANAS - positive	-0.003692607	0.400	-0.012290264	0.004905049	50275
IL-6R (ahluwalia-cis)	Identify Task – reaction time	-0.007388214	0.139	-0.017182749	0.00240632	31248
IL-6R (ahluwalia-cis)	Detect Task – reaction time	-0.002237753	0.662	-0.012257602	0.007782095	30872
IL-6R (ahluwalia-cis)	OCL Task - accuracy	-0.005819009	0.285	-0.016485395	0.004847377	32182
IL-6R (ahluwalia-cis)	One-back Task - accuracy	0.001228477	0.821	-0.00942373	0.011880684	31805
IL-6R (ahluwalia-cis)	RFFT	-0.007359532	0.162	-0.017685313	0.002966249	32250
IL-6R (ahluwalia-cis)	PANAS – negative	0.004652218	0.281	-0.003801535	0.01310597	50275
IL-6R (ahluwalia-cis)	PANAS - positive	-0.003825848	0.383	-0.012426959	0.004775262	50275
sIL6R (rosa)	Identify Task – reaction time	-0.006482964	0.194	-0.016265604	0.003299675	31248
sIL6R (rosa)	Detect Task – reaction time	-0.000565435	0.912	-0.01057052	0.00943965	30872
sIL6R (rosa)	OCL Task - accuracy	-0.012981218	0.017	-0.023632487	-0.00232995	32182
sIL6R (rosa)	One-back Task - accuracy	-0.005672935	0.296	-0.01631509	0.00496922	31805
sIL6R (rosa)	RFFT	-0.001518224	0.773	-0.011827491	0.008791043	32250
sIL6R (rosa)	PANAS – negative	0.005268752	0.222	-0.00318464	0.013722145	50275
sIL6R (rosa)	PANAS - positive	-0.003589323	0.413	-0.012190103	0.005011458	50275
IL-6R (sarwar)	Identify Task – reaction time	-0.005229929	0.294	-0.015001331	0.004541473	31248
IL-6R (sarwar)	Detect Task – reaction time	-0.002107915	0.679	-0.012098351	0.007882521	30872

IL-6R (sarwar)	OCL Task - accuracy	-0.008406874	0.121	-0.019045513	0.002231766	32182
IL-6R (sarwar)	One-back Task - accuracy	0.001261055	0.816	-0.009362978	0.011885087	31805
IL-6R (sarwar)	RFFT	-0.00224542	0.670	-0.01256145	0.008070611	32250
IL-6R (sarwar)	PANAS – negative	0.004621141	0.284	-0.003829182	0.013071463	50275
IL-6R (sarwar)	PANAS - positive	-0.007908285	0.071	-0.016505693	0.000689122	50275
CRP (said-genome-wide)	Identify Task – reaction time	-0.004384599	0.379	-0.014157457	0.00538826	31248
CRP (said-genome-wide)	Detect Task – reaction time	0.005849798	0.251	-0.004130549	0.015830146	30872
CRP (said-genome-wide)	OCL Task - accuracy	0.006495752	0.231	-0.004134355	0.017125859	32182
CRP (said-genome-wide)	One-back Task - accuracy	0.006311614	0.244	-0.00430412	0.016927349	31805
CRP (said-genome-wide)	RFFT	0.005411245	0.306	-0.004946475	0.015768965	32250
CRP (said-genome-wide)	PANAS – negative	-0.004457444	0.302	-0.012925397	0.004010509	50275
CRP (said-genome-wide)	PANAS - positive	-0.000471111	0.915	-0.009086725	0.008144503	50275
IL-6 (ahluwalia-genome-wide)	Identify Task – reaction time	-0.007394591	0.141	-0.017231994	0.002442813	31248
IL-6 (ahluwalia-genome-wide)	Detect Task – reaction time	-0.003216338	0.531	-0.013280611	0.006847936	30872
IL-6 (ahluwalia-genome-wide)	OCL Task - accuracy	-0.004718069	0.388	-0.015431196	0.005995059	32182
IL-6 (ahluwalia-genome-wide)	One-back Task - accuracy	0.001088414	0.842	-0.009610476	0.011787304	31805
IL-6 (ahluwalia-genome-wide)	RFFT	-0.007272177	0.169	-0.017646042	0.003101689	32250
IL-6 (ahluwalia-genome-wide)	PANAS – negative	0.004892038	0.259	-0.003599313	0.013383388	50275
IL-6 (ahluwalia-genome-wide)	PANAS - positive	-0.001706063	0.699	-0.010345489	0.006933363	50275

Table 11. Standardised GRS on continuous outcomes (2nd degree relatives removed)

Standardised GRS	Standardised Outcomes	Beta	p-value	LCI	UCI	N
CRP (said-cis)	Identify Task – reaction time	-0.004455903	0.375	-0.014297241	0.005385435	30885
CRP (said-cis)	Detect Task – reaction time	0.006944798	0.177	-0.003128157	0.017017752	30514
CRP (said-cis)	OCL Task - accuracy	-0.006845139	0.211	-0.017576303	0.003886025	31806
CRP (said-cis)	One-back Task - accuracy	-0.003839799	0.482	-0.014552201	0.006872603	31430
CRP (said-cis)	RFFT	0.006171204	0.243	-0.004197376	0.016539785	31862
CRP (said-cis)	PANAS – negative	0.009857729	0.023	0.001341303	0.018374155	49586
CRP (said-cis)	PANAS - positive	0.005861774	0.184	-0.002791759	0.014515308	49586
GlycA (borges)	Identify Task – reaction time	0.001265473	0.801	-0.008551323	0.01108227	30885
GlycA (borges)	Detect Task – reaction time	0.002739723	0.593	-0.007313541	0.012792988	30514
GlycA (borges)	OCL Task - accuracy	0.004528117	0.407	-0.006177087	0.015233321	31806
GlycA (borges)	One-back Task - accuracy	0.001261024	0.817	-0.009430819	0.011952867	31430
GlycA (borges)	RFFT	-0.004472728	0.398	-0.014855337	0.00590988	31862
GlycA (borges)	PANAS – negative	0.012289134	0.005	0.003775534	0.020802734	49586
GlycA (borges)	PANAS - positive	-0.00339255	0.442	-0.012043564	0.005258464	49586
IL-6R (ahluwalia-cis)	Identify Task – reaction time	-0.006629579	0.187	-0.01647197	0.003212811	30885
IL-6R (ahluwalia-cis)	Detect Task – reaction time	-0.000922534	0.858	-0.011008193	0.009163125	30514
IL-6R (ahluwalia-cis)	OCL Task - accuracy	-0.005376679	0.326	-0.016116486	0.005363128	31806
IL-6R (ahluwalia-cis)	One-back Task - accuracy	0.001617299	0.767	-0.009098969	0.012333567	31430
IL-6R (ahluwalia-cis)	RFFT	-0.007750897	0.144	-0.018146283	0.002644489	31862
IL-6R (ahluwalia-cis)	PANAS – negative	0.004544888	0.296	-0.003972594	0.01306237	49586
IL-6R (ahluwalia-cis)	PANAS - positive	-0.003255724	0.461	-0.011910083	0.005398635	49586
sIL6R (rosa)	Identify Task – reaction time	-0.005873464	0.242	-0.015704021	0.003957093	30885
sIL6R (rosa)	Detect Task – reaction time	0.000973275	0.850	-0.009097362	0.011043913	30514
sIL6R (rosa)	OCL Task - accuracy	-0.012447692	0.023	-0.023172184	-0.001723201	31806
sIL6R (rosa)	One-back Task - accuracy	-0.005575441	0.307	-0.016281637	0.005130756	31430
sIL6R (rosa)	RFFT	-0.002065713	0.697	-0.012447815	0.008316389	31862
sIL6R (rosa)	PANAS – negative	0.005671932	0.192	-0.00284578	0.014189644	49586
sIL6R (rosa)	PANAS - positive	-0.003427152	0.438	-0.012081793	0.00522749	49586
IL-6R (sarwar)	Identify Task – reaction time	-0.004503529	0.369	-0.014321039	0.005313981	30885
IL-6R (sarwar)	Detect Task – reaction time	-0.000572008	0.911	-0.010626347	0.009482331	30514

IL-6R (sarwar)	OCL Task - accuracy	-0.007931195	0.147	-0.018641834	0.002779444	31806
IL-6R (sarwar)	One-back Task - accuracy	0.001561761	0.775	-0.00912495	0.012248472	31430
IL-6R (sarwar)	RFFT	-0.002749847	0.604	-0.01313688	0.007637186	31862
IL-6R (sarwar)	PANAS – negative	0.004408061	0.310	-0.004106041	0.012922162	49586
IL-6R (sarwar)	PANAS - positive	-0.007323901	0.097	-0.015974627	0.001326825	49586
CRP (said-genome-wide)	Identify Task – reaction time	-0.00441728	0.377	-0.014226265	0.005391706	30885
CRP (said-genome-wide)	Detect Task – reaction time	0.006311434	0.218	-0.003722837	0.016345706	30514
CRP (said-genome-wide)	OCL Task - accuracy	0.006649428	0.223	-0.004041917	0.017340774	31806
CRP (said-genome-wide)	One-back Task - accuracy	0.005929476	0.276	-0.004738813	0.016597765	31430
CRP (said-genome-wide)	RFFT	0.004949002	0.352	-0.005479586	0.01537759	31862
CRP (said-genome-wide)	PANAS – negative	-0.004004421	0.358	-0.012537485	0.004528644	49586
CRP (said-genome-wide)	PANAS - positive	-0.000744121	0.866	-0.009414337	0.007926095	49586
IL-6 (ahluwalia-genome-wide)	Identify Task – reaction time	-0.006600991	0.191	-0.016486541	0.00328456	30885
IL-6 (ahluwalia-genome-wide)	Detect Task – reaction time	-0.001896891	0.714	-0.012027463	0.008233682	30514
IL-6 (ahluwalia-genome-wide)	OCL Task - accuracy	-0.004187567	0.447	-0.014974478	0.006599344	31806
IL-6 (ahluwalia-genome-wide)	One-back Task - accuracy	0.001494971	0.785	-0.009268274	0.012258216	31430
IL-6 (ahluwalia-genome-wide)	RFFT	-0.007700978	0.148	-0.018144867	0.002742911	31862
IL-6 (ahluwalia-genome-wide)	PANAS – negative	0.004759201	0.276	-0.0037966	0.013315002	49586
IL-6 (ahluwalia-genome-wide)	PANAS - positive	-0.001113178	0.802	-0.009806523	0.007580167	49586

Table 12. Standardised GRS on continuous outcomes (3rd degree relatives removed)

Standardised GRS	Standardised Outcomes	Beta	p-value	LCI	UCI	N
CRP (said-cis)	Identify Task – reaction time	-0.002774482	0.586	-0.012749699	0.007200734	30055
CRP (said-cis)	Detect Task – reaction time	0.007615498	0.143	-0.002576949	0.017807945	29688
CRP (said-cis)	OCL Task - accuracy	-0.007644057	0.168	-0.018519803	0.003231689	30948
CRP (said-cis)	One-back Task - accuracy	-0.003689577	0.506	-0.014557446	0.007178292	30581
CRP (said-cis)	RFFT	0.006567816	0.220	-0.003937313	0.017072945	30978
CRP (said-cis)	PANAS – negative	0.010495546	0.017	0.001849709	0.019141384	48230
CRP (said-cis)	PANAS - positive	0.005831615	0.193	-0.002940174	0.014603405	48230
GlycA (borges)	Identify Task – reaction time	-0.000180337	0.972	-0.010122833	0.009762159	30055
GlycA (borges)	Detect Task – reaction time	0.002597782	0.617	-0.007573292	0.012768857	29688
GlycA (borges)	OCL Task - accuracy	0.004564767	0.409	-0.006281792	0.015411325	30948
GlycA (borges)	One-back Task - accuracy	0.000830935	0.881	-0.010012673	0.011674543	30581
GlycA (borges)	RFFT	-0.005200444	0.332	-0.015716549	0.00531566	30978
GlycA (borges)	PANAS – negative	0.010745678	0.015	0.00209993	0.019391427	48230
GlycA (borges)	PANAS - positive	-0.003318834	0.458	-0.012090663	0.005452995	48230
IL-6R (ahluwalia-cis)	Identify Task – reaction time	-0.00691165	0.174	-0.016878898	0.003055598	30055
IL-6R (ahluwalia-cis)	Detect Task – reaction time	-0.000272639	0.958	-0.010472643	0.009927365	29688
IL-6R (ahluwalia-cis)	OCL Task - accuracy	-0.004406893	0.427	-0.01528395	0.006470165	30948
IL-6R (ahluwalia-cis)	One-back Task - accuracy	0.001232496	0.824	-0.009631128	0.012096121	30581
IL-6R (ahluwalia-cis)	RFFT	-0.006775222	0.207	-0.017307765	0.003757322	30978
IL-6R (ahluwalia-cis)	PANAS – negative	0.005925359	0.179	-0.002721924	0.014572643	48230
IL-6R (ahluwalia-cis)	PANAS - positive	-0.003399121	0.448	-0.012172129	0.005373886	48230
sIL6R (rosa)	Identify Task – reaction time	-0.006150564	0.226	-0.016099049	0.00379792	30055
sIL6R (rosa)	Detect Task – reaction time	0.001400574	0.787	-0.008776395	0.011577544	29688
sIL6R (rosa)	OCL Task - accuracy	-0.011708759	0.035	-0.022564667	-0.000852851	30948
sIL6R (rosa)	One-back Task - accuracy	-0.00587703	0.288	-0.016724268	0.004970208	30581
sIL6R (rosa)	RFFT	-0.000927542	0.863	-0.011439615	0.009584532	30978
sIL6R (rosa)	PANAS – negative	0.006161837	0.162	-0.002482684	0.014806357	48230
sIL6R (rosa)	PANAS - positive	-0.003788907	0.397	-0.012559112	0.004981298	48230
IL-6R (sarwar)	Identify Task – reaction time	-0.005075206	0.317	-0.01501438	0.004863967	30055
IL-6R (sarwar)	Detect Task – reaction time	-0.000128678	0.980	-0.010293975	0.010036619	29688

IL-6R (sarwar)	OCL Task - accuracy	-0.005936317	0.283	-0.016781671	0.004909036	30948
IL-6R (sarwar)	One-back Task - accuracy	0.001771228	0.749	-0.009060039	0.012602496	30581
IL-6R (sarwar)	RFFT	-0.00101124	0.851	-0.011533065	0.009510584	30978
IL-6R (sarwar)	PANAS – negative	0.005588038	0.205	-0.00305536	0.014231436	48230
IL-6R (sarwar)	PANAS - positive	-0.007725396	0.084	-0.016494226	0.001043433	48230
CRP (said-genome-wide)	Identify Task – reaction time	-0.003617212	0.475	-0.013545849	0.006311426	30055
CRP (said-genome-wide)	Detect Task – reaction time	0.006502643	0.209	-0.003633692	0.016638977	29688
CRP (said-genome-wide)	OCL Task - accuracy	0.005456883	0.323	-0.005364119	0.016277884	30948
CRP (said-genome-wide)	One-back Task - accuracy	0.004386723	0.426	-0.00642168	0.015195127	30581
CRP (said-genome-wide)	RFFT	0.004065849	0.450	-0.006483806	0.014615503	30978
CRP (said-genome-wide)	PANAS – negative	-0.002859669	0.517	-0.011515421	0.005796083	48230
CRP (said-genome-wide)	PANAS - positive	-0.000701674	0.876	-0.009483198	0.00807985	48230
IL-6 (ahluwalia-genome-wide)	Identify Task – reaction time	-0.006858244	0.179	-0.016872277	0.00315579	30055
IL-6 (ahluwalia-genome-wide)	Detect Task – reaction time	-0.001390068	0.790	-0.011638639	0.008858503	29688
IL-6 (ahluwalia-genome-wide)	OCL Task - accuracy	-0.003063095	0.583	-0.013990921	0.00786473	30948
IL-6 (ahluwalia-genome-wide)	One-back Task - accuracy	0.001092963	0.844	-0.009821351	0.012007278	30581
IL-6 (ahluwalia-genome-wide)	RFFT	-0.006651944	0.218	-0.017237636	0.003933748	30978
IL-6 (ahluwalia-genome-wide)	PANAS – negative	0.006212709	0.161	-0.002475698	0.014901116	48230
IL-6 (ahluwalia-genome-wide)	PANAS - positive	-0.001142555	0.799	-0.009957346	0.007672235	48230

SUPPLEMENTARY FIGURES

Figure 1. Flow diagram of total sample size available for genetic risk score analysis.

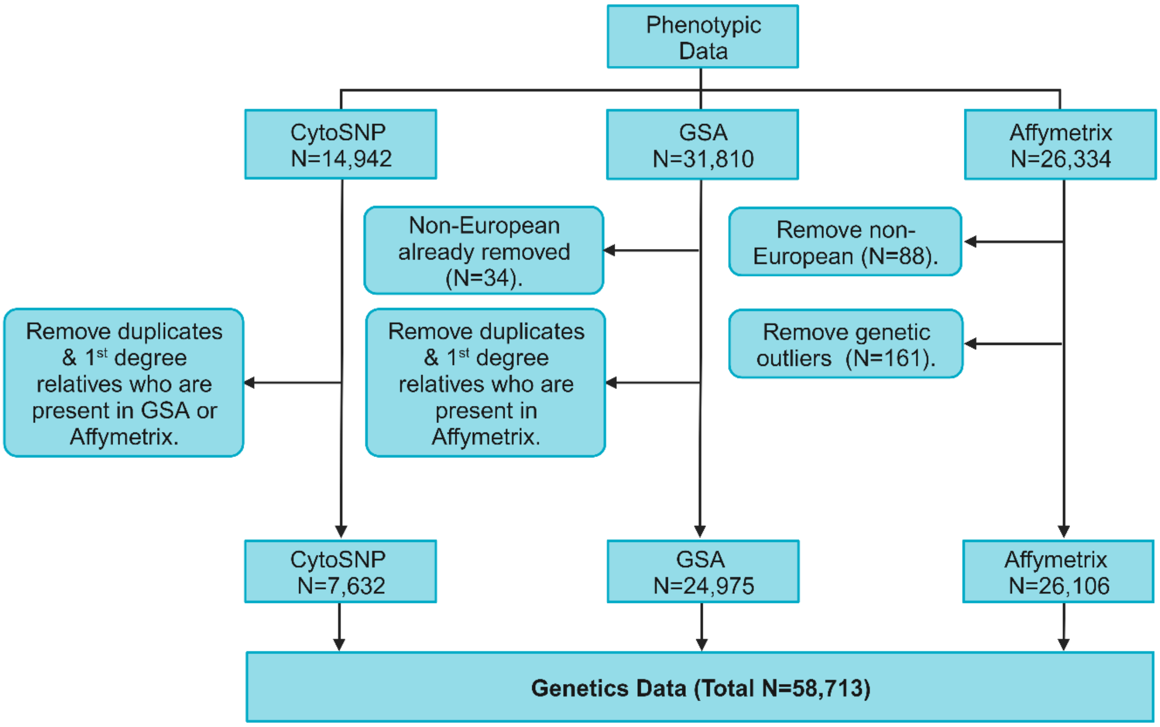

Note: Phenotypic data includes individuals in Lifelines who are ≥ 18 years and excludes individuals who self-report one of the following health conditions: Alzheimer's disease, dementia, epilepsy, multiple sclerosis, Parkinson's disease, stroke. Duplicates and first-degree relatives were removed from CytoSNP where possible due to its poorer imputation quality in comparison to the other chips. Lists of duplicates and first-degree relatives to remove between chips were provided by Lifelines. Genetic outliers and non-European individuals were also provided by Lifelines. Figure created using *Biorender.com*.

References

1. Ligthart, S., Vaez, A., Võsa, U., Stathopoulou, M. G., de Vries, P. S., Prins, B. P., Van der Most, P. J., Tanaka, T., Naderi, E., Rose, L. M., *et al.* Genome Analyses of >200,000 Individuals Identify 58 Loci for Chronic Inflammation and Highlight Pathways that Link Inflammation and Complex Disorders. *Am. J. Hum. Genet.* **103**, 691–706 (2018).

2. Ahluwalia, T. S., Prins, B. P., Abdollahi, M., Armstrong, N. J., Aslibekyan, S., Bain, L., Jefferis, B., Baumert, J., Beekman, M., Ben-Shlomo, Y., *et al.* Genome-wide association study of circulating interleukin 6 levels identifies novel loci. *Hum. Mol. Genet.* **30**, 393–409 (2021).

3. Sarwar, N., Butterworth, A. S., Freitag, D. F., Gregson, J., Willeit, P., Gorman, D. N., Gao, P., Saleheen, D., Rendon, A., Nelson, C. P., *et al.* Interleukin-6 receptor pathways in coronary heart disease: A collaborative meta-analysis of 82 studies. *Lancet* **379**, 1205–1213 (2012).

4. Ferreira, R. C., Freitag, D. F., Cutler, A. J., Howson, J. M. M., Rainbow, D. B., Smyth, D. J., Kaptoge, S., Clarke, P., Boreham, C., Coulson, R. M., *et al.* Functional IL6R 358Ala Allele Impairs Classical IL-6 Receptor Signaling and Influences Risk of Diverse Inflammatory Diseases. *PLOS Genet.* **9**, e1003444 (2013).

5. Galicia, J. C., Tai, H., Komatsu, Y., Shimada, Y., Akazawa, K. & Yoshie, H. Polymorphisms in the IL-6 receptor (IL-6R) gene: strong evidence that serum levels of soluble IL-6R are genetically influenced. *Genes Immun.* **5**, 513–516 (2004).

6. Calabrese, L. H. & Rose-John, S. IL-6 biology: implications for clinical targeting in rheumatic disease. *Nat. Rev. Rheumatol.* **10**, 720–727 (2014).

7. Rosa, M., Chignon, A., Li, Z., Boulanger, M. C., Arsenault, B. J., Bossé, Y., Thériault, S. & Mathieu, P. A Mendelian randomization study of IL6 signaling in cardiovascular diseases, immune-related disorders and longevity. *npj Genomic Med.* **4**, 1–10 (2019).

8. Sun, B. B., Maranville, J. C., Peters, J. E., Stacey, D., Staley, J. R., Blackshaw, J., Burgess, S., Jiang, T., Paige, E., Surendran, P., *et al.* Genomic atlas of the human plasma proteome. *Nature* **558**, 73–79 (2018).

9. Aulchenko, Y. S., De Koning, D. J. & Haley, C. Genomewide Rapid Association Using Mixed Model and Regression: A Fast and Simple Method For Genomewide Pedigree-Based Quantitative Trait Loci Association Analysis. *Genetics* **177**, 577–585 (2007).

10. Yang, J., Lee, S. H., Goddard, M. E. & Visscher, P. M. GCTA: A Tool for Genome-wide Complex Trait Analysis. *Am. J. Hum. Genet.* **88**, 76 (2011).

11. Manichaikul, A., Mychaleckyj, J. C., Rich, S. S., Daly, K., Sale, M. & Chen, W. M. Robust relationship inference in genome-wide association studies. *Bioinformatics* **26**, 2867–2873 (2010).

12. Chang, C. C., Chow, C. C., Tellier, L. C. A. M., Vattikuti, S., Purcell, S. M. & Lee, J. J. Second-generation PLINK: Rising to the challenge of larger and richer datasets. *Gigascience* **4**, 7 (2015).

13. Chang, C. & Purcell, S. PLINK version 2. www.cog-genomics.org/plink/2.0/.

14. Burgess, S. & Thompson, S. G. Avoiding bias from weak instruments in Mendelian randomization studies. *Int. J. Epidemiol.* **40**, 755–764 (2011).

15. Staiger, D. & Stock, J. H. Instrumental Variables Regression with Weak Instruments. *Econometrica* **65**, 557 (1997).

16. Slaney, C., Sallis, H. M., Jones, H. J., Dardani, C., Tilling, K., Munafò, M. R., Davey Smith, G., Mahedy, L. & Khandaker, G. M. Association between inflammation and cognition: Triangulation of evidence using a population-based cohort and Mendelian randomization analyses. *Brain. Behav. Immun.* **110**, 30–42 (2023).

17. Khandaker, G. M., Zuber, V., Rees, J. M. B., Carvalho, L., Mason, A. M., Foley, C. N., Gkatzionis, A., Jones, P. B. & Burgess, S. Shared mechanisms between coronary heart disease and depression: findings from a large UK general population-based cohort. *Mol. Psychiatry* **25**, 1477–1486 (2020).

18. Wium-Andersen, M. K., Oørsted, D. D. & Nordestgaard, B. G. Elevated C-reactive protein, depression, somatic diseases, and all-cause mortality: a mendelian randomization study. *Biol. Psychiatry* **76**, 249–257 (2014).

19. Ye, Z., Kappelmann, N., Moser, S., Davey Smith, G., Burgess, S., Jones, P. B. & Khandaker, G. M. Role of inflammation in depression and anxiety: Tests for disorder specificity, linearity and potential causality of association in the UK Biobank. *EClinicalMedicine* **38**, (2021).

20. Bekkevold, O. J., Damås, J. K., Brumpton, B. M. & Åsvold, B. O. The causal role of C-reactive protein and interleukin-6 on anxiety and depression symptoms and life satisfaction: Mendelian randomisation analyses in the HUNT study. *Psychol. Med.* **52**, (2023).

21. Galan, D., Perry, B. I., Warrier, V., Davidson, C. C., Stupart, O., Easton, D., Khandaker, G. M. & Murray, G. K. Applying Mendelian randomization to appraise causality in relationships between smoking, depression and inflammation. *Sci. Reports 2022 121* **12**, 1–12 (2022).
